# Perceptions of the 2011 ACGME duty hour requirements among residents in all core programs at a large academic medical center

**DOI:** 10.1186/s12909-017-1033-x

**Published:** 2017-11-10

**Authors:** Benjamin J. Sandefur, Diana M. Shewmaker, Christine M. Lohse, Steven H. Rose, James E. Colletti

**Affiliations:** 1 Department of Emergency Medicine, Mayo Clinic, 200 First St SW, Rochester, MN 55905 USA; 2 Division of Biomedical Statistics and Informatics, Mayo Clinic, Rochester, MN USA; 3 Department of Anesthesiology, Mayo Clinic, Rochester, MN USA

**Keywords:** Duty hour, Fatigue, Medical education, Medical error, Resident perception, Work hour

## Abstract

**Background:**

The Accreditation Council for Graduate Medical Education (ACGME) implemented revisions to resident duty hour requirements (DHRs) in 2011 to improve patient safety and resident well-being. Perceptions of DHRs have been reported to vary by training stage and specialty among internal medicine and general surgery residents. The authors explored perceptions of DHRs among all residents at a large academic medical center.

**Methods:**

The authors administered an anonymous cross-sectional survey about DHRs to residents enrolled in all ACGME-accredited core residency programs at their institution. Residents were categorized as *medical and pediatric*, *surgery*, or *other*.

**Results:**

In total, 736 residents representing 24 core specialty residency programs were surveyed. The authors received responses from 495 residents (67%). A majority reported satisfaction (78%) with DHRs and believed DHRs positively affect their training (73%). Residents in surgical specialties and in advanced stages of training were significantly less likely to view DHRs favorably. Most respondents believed fatigue contributes to errors (89%) and DHRs reduce both fatigue (80%) and performance of clinical duties while fatigued (74%). A minority of respondents (37%) believed that DHRs decrease medical errors. This finding may reflect beliefs that handovers contribute more to errors than fatigue (41%). Negative perceived effects included diminished patient familiarity and continuity of care (62%) and diminished clinical educational experiences for residents (41%).

**Conclusions:**

A majority of residents reported satisfaction with the 2011 DHRs, although satisfaction was significantly less among residents in surgical specialties and those in advanced stages of training.

## Background

In 1999, the Institute of Medicine released a landmark report, “To Err Is Human: Building a Safer Health System,” which implicated medical errors as a principal cause of patient morbidity and mortality [1]. The report concluded that deaths due to preventable medical errors exceeded deaths attributed to motor vehicle accidents, breast cancer, or acquired immunodeficiency syndrome. In 2003, the Accreditation Council for Graduate Medical Education (ACGME) instituted duty hour requirements (DHRs) as a component of common standards governing resident education, with the goal of improving patient safety and resident well-being. The standards required a limit of 80 weekly work hours averaged over 4 weeks, a limit on continuous duty of 24 h with an additional 6 h for transfers of care, 1 day off in every 7, and a limit on in-house call to once in every 3 nights [2, 3]. In 2008, the Institute of Medicine published “Resident Duty Hours: Enhancing Sleep, Supervision and Safety,” which recommended enhanced monitoring of duty hours, regulation of resident caseloads, enhanced supervision, and improved transitions of patient care, or *handovers*, and simultaneously acknowledged an absence of crucial research investigations [4]. The ACGME implemented revised standards in 2011 to more closely regulate duty hours and trainee supervision. Among the new regulations were a limit of 16 contiguous work hours for first-year trainees, a limit of 4 h for transfer of care after a 24-h work period, a recommendation of 10 h off duty (required 8 h off duty) between duty periods, and requirements for improved handovers of care and supervision of residents [5].

Performance impairment due to sleep deprivation and fatigue is well established [6–8]. However, the effects of the ACGME DHRs on resident well-being and education, as well as patient safety, continue to be debated. Some studies have suggested that DHRs are beneficial for alertness [9] and are supported by residents [10, 11]; other studies have raised concerns about adverse effects on clinical training [12, 13] and overall quality of patient care [13, 14]. One structured review of the empirical literature on duty hour limits and patient care and resident outcomes found that existing studies assessing safety and quality in teaching institutions have yielded heterogeneous results [15]. Studies demonstrating positive effects of DHRs were more likely to have been conducted in medical specialties, while those conducted within surgical disciplines raised concern about the negative effects of DHRs on patient safety and continuity of care [15].

Determining the impact of DHRs on a complex system of medical education and health care delivery continues to be an important area of scholarship. Improving our understanding of residents’ attitudes and opinions about DHRs may provide valuable insights. Philibert [16] reported that programs in which residents view duty hour compliance as a strength are characterized as more efficient, collegial, and responsive to problems. Existing literature suggests that there may be heterogeneity in perceptions of DHRs among the specialties and the stages of training, as highlighted by a recent study demonstrating that internal medicine respondents favored DHRs more than general surgery respondents among a subset analysis of 49 respondents at 1 institution. In addition, in this study, first-year residents viewed DHRs in a more positive manner than senior residents and faculty [17].

Our anecdotal experience from a large academic medical center with 736 residents enrolled in 24 ACGME-accredited core residency programs suggests that residents generally view DHRs positively, though with coinciding concerns over lost educational opportunities. In this study, we aimed to explore resident satisfaction with the ACGME DHRs and perceptions of the impact of DHRs on patient care, their education, fatigue mitigation, and medical errors. We also aimed to explore differences in satisfaction with DHRs and perceptions of the overall effects of DHRs on training by specialty type and stage of training. We hypothesized that significant differences in perceptions of DHRs exist between surgical and nonsurgical specialties, as well as junior and senior residents.

## Methods

All residents currently enrolled in ACGME-accredited core residency programs at Mayo Clinic’s campus in Rochester, Minnesota were eligible to participate in our cross-sectional survey. The Mayo Clinic Institutional Review Board approved the study.

We collected data from August 11 through October 1, 2014. Residents were invited through email to complete an anonymous electronic survey that included an informed consent statement on the first page. An email reminder was distributed midway through the survey period. Participation was voluntary, and residents received no incentive to participate. Study data were collected and managed using REDCap (Research Electronic Data Capture) electronic data capture tools hosted at Mayo Clinic. REDCap is a secure, Web-based application designed to support data capture for research studies [18].

We asked residents to provide information related to their demographic characteristics; satisfaction with and attitudes about DHRs; perceptions of culture and DHRs; perceptions of fatigue, medical errors, and relation to DHRs; and perceptions of the educational effect of DHRs. No individual survey questions were mandatory. We asked participants to record their specialty, and they were informed that specialty data would be reported only in aggregate specialty groupings. Data were not reported individually for core residencies, because the program size for some specialties was too small to ensure respondent confidentiality if reported independently.

Residency programs were categorized into 3 specialty groups for comparison: medical and pediatric (*med/peds*) (ie, internal medicine and subspecialties and pediatric and adolescent medicine); surgical (*surgery*) (ie, anesthesiology, dental specialties, neurologic surgery, obstetrics and gynecology, ophthalmology, orthopedic surgery, otorhinolaryngology, surgery and subspecialties, and urology); and other (*other*) (ie, dermatology, emergency medicine, family medicine, genetics, neurology, pathology and laboratory medicine, physical medicine and rehabilitation, psychiatry, radiation oncology, radiology, sports medicine, transitional year, and other). This categorization represents the subspecialty grouping applied by the Mayo School of Graduate Medical Education at Mayo Clinic in Minnesota. Residents were categorized as *junior* if they were in their first or second postgraduate year (PGY) and *senior* if they were in or beyond their third PGY.

We summarized survey responses using frequency counts and percentages. Percentages were calculated using the total number of nonmissing responses obtained for an individual question. Comparisons between junior and senior residents and among specialty types regarding satisfaction with DHRs and effect on training were evaluated with Wilcoxon rank sum and Kruskal-Wallis tests. Cronbach α was calculated for key survey questions that addressed similar concepts. Statistical analyses were performed with statistical software (version 9.3; SAS Institute Inc). *P* < .05 was considered statistically significant.

The reliability, validity, and psychometric properties of our instrument were addressed through a multifaceted approach. We conducted a literature review to identify existing survey research assessing resident perspectives on DHRs, which informed the construction of our survey instrument. A portion of our instrument was adapted from a previously published survey that targeted a national population of neurosurgical residents [12]. To address content validity, a multidisciplinary group of medical educators (in the specialties anesthesiology, emergency medicine, general surgery, internal medicine, obstetrics and gynecology, pediatrics, and psychiatry) reviewed and edited our survey questions. A pilot survey was conducted among these medical educators to eliminate questions that could be misinterpreted or could show bias. Pilot survey respondents provided feedback on participant solicitation and consent, content, question-and-answer wording, and the methodology of survey administration.

## Results

### Demographic characteristics of respondents

We identified 736 residents who met study criteria and invited them to participate in our survey. We received 495 responses (response rate, 67%), of which 382 surveys (77%) were completed in entirety. Residents from PGY-1 through PGY-6 and above were represented, and junior residents comprised 36% of respondents. Women comprised 40% of our cohort, and 66% of respondents were married. Of the 439 respondents (89%) who provided a specialty, 131 (30%) were categorized as med/peds, 151 (34%) as surgery, and 157 (36%) as other. Table 1 summarizes the respondent demographic data.

**Table 1:** Demographic Characteristics of the 495 Survey Respondents

Survey Question	Respondents, No. (%)
Age, y (*n* = 441)	
20–24	2 (<1)
25–29	202 (46)
30–34	179 (41)
35–39	34 (8)
40–44	9 (2)
45–50	8 (2)
> 50	7 (2)
Sex (*n* = 440)	
Female	176 (40)
Male	264 (60)
Marital status (*n* = 441)	
Married/domestic partner	291 (66)
Living with significant other	34 (8)
Divorced	8 (2)
Single, never married	108 (24)
Children (*n* = 440)	
Yes	161 (37)
No	279 (63)
Medical school (*n* = 437)	
US medical graduate	377 (86)
Foreign medical graduate	60 (14)
Anticipated practice type (*n* = 440)	
Academic	219 (50)
Private	74 (17)
Unsure	147 (33)
PGY (*n* = 438)	
1	76 (17)
2	81 (18)
3	97 (22)
4	62 (14)
5	56 (13)
≥6	66 (15)
Specialty, No. of respondents (% of respondents; % of specialty responding) (*n* = 439)	
Anesthesiology	48 (11; 81)
Dermatology	9 (2; 36)
Emergency medicine	20 (5; 80)
Family medicine	17 (4; 68)
Genetics	1 (<1; 50)
Internal medicine and subspecialties	105 (24; 61)
Neurologic surgery	9 (2; 41)
Neurology	18 (4; 67)
Obstetrics and gynecology	14 (3; 82)
Ophthalmology	4 (1; 33)
Orthopedic surgery	31 (7; 53)
Otorhinolaryngology	2 (<1; 10)
Pathology and laboratory medicine	14 (3; 56)
Pediatric and adolescent medicine	25 (6; 60)
Physical medicine and rehabilitation	15 (3; 65)
Psychiatry	19 (4; 54)
Radiation oncology	6 (1; 50)
Radiology	24 (5; 46)
Surgery and subspecialties	34 (8; 34)
Urology	9 (2; 45)
Other	15 (3)

*Abbreviation*: *PGY* postgraduate year

### Satisfaction with and attitudes about duty hours

A majority of residents (78%) reported satisfaction with the ACGME DHRs and reported that the requirements affect their training in an overall positive manner (73%) (Cronbach α=.70). Junior residents were significantly more likely (*P* ≤ .001) than senior residents to report satisfaction with DHRs (84% vs 74%) and an overall positive effect on their training (85% vs 66%). Residents in surgical specialties were significantly less likely (*P* ≤ .001) to report satisfaction with DHRs (66%) or an overall positive effect on training (63%) than residents in med/peds (87% and 76%) or other (82% and 81%) specialties (Table 2).

**Table 2:** Resident Satisfaction and Comparison by PGY and Specialty

Survey Question	Possible Survey Response			
	Strongly agree	Agree	Disagree	Strongly disagree
I am satisfied with the current duty hour rules (*n* = 391)	101 (26)	204 (52)	62 (16)	24 (6)
	Strongly positive	Positive	Negative	Strongly negative
Overall, I believe the current ACGME standards affect my training in a _________ way (*n* = 388)	34 (9)	249 (64)	89 (23)	16 (4)
I am satisfied with the current duty hour rules^a,b^	Strongly agree	Agree	Disagree	Strongly disagree
PGY 1 and PGY 2 (*n* = 144)	48 (33)	74 (51)	18 (13)	4 (3)
PGY 3 and greater (*n* = 244)	51 (21)	129 (53)	44 (18)	20 (8)
Med/peds (*n* = 119)	38 (32)	65 (55)	14 (12)	2 (2)
Surgery (*n* = 140)	20 (14)	73 (52)	32 (23)	15 (11)
Other (*n* = 130)	42 (32)	65 (50)	16 (12)	7 (5)
Overall, I believe the current ACGME standards affect my training in a _________ way^c,d^	Strongly positive	Positive	Negative	Strongly negative
PGY 1 and PGY 2 (*n* = 144)	16 (11)	106 (74)	19 (13)	3 (2)
PGY 3 and beyond (*n* = 241)	16 (7)	143 (59)	69 (29)	13 (5)
Med/peds (*n* = 118)	11 (9)	79 (67)	26 (22)	2 (2)
Surgery (*n* = 138)	9 (7)	77 (56)	39 (28)	13 (9)
Other (n = 130)	13 (10)	92 (71)	24 (18)	1 (1)

*Abbreviations*: *ACGME* Accreditation Council for Graduate Medical Education, *med*/*peds*, medical and pediatric, *PGY* postgraduate year

^a^Comparison by satisfaction table for PGY 1 + 2 vs PGY≥3, *P* = .001

^b^Comparison by satisfaction table for Med/peds vs Surg vs Other, *P* < .001

^c^Comparison by effect on training table for PGY 1 + 2 vs PGY≥3, *P* < .001

^d^Comparison by effect on training table for Med/peds vs Surg vs Other, *P* = .001

### Perceptions of culture and duty hour violations

Most respondents believed that DHRs were respected within their institution (93%), are enforced (95%), and were reflected in their work schedule (94%) (Cronbach α=.92) (Table 3). Some residents reported exceeding the 28-h maximum duty hour requirement (8%) or the 80-h weekly maximum duty hour limit (11%) more than rarely within the past year. Many residents believed that there are situations when a violation of DHRs may be justified (80%). Few reported having been specifically asked to violate duty hour rules (6%) either by a peer (*n* = 2), a senior resident (*n* = 7), an attending physician (*n* = 12), or some other person (*n* = 5).

**Table 3:** Resident Perceptions of Culture and Duty Hour Violations

Survey Question	Response, No. (%)
The ACGME duty hour rules are respected at my institution (*n* = 390)	
Strongly agree	235 (60)
Agree	128 (33)
Disagree	21 (5)
Strongly disagree	6 (2)
The ACGME duty hour rules are consistently reflected in my work schedule (*n* = 390)	
Strongly agree	224 (57)
Agree	143 (37)
Disagree	19 (5)
Strongly disagree	4 (1)
The ACGME duty hour rules are enforced at my institution (*n* = 390)	
Strongly agree	235 (60)
Agree	134 (34)
Disagree	16 (4)
Strongly disagree	5 (1)
During the past year, I have exceeded the 28-h maximum work hour requirement (24 h continuous duty +4 h for transition of care) (*n* = 390)	
Frequently	10 (3)
Occasionally	22 (6)
Rarely	56 (14)
Never	302 (77)
During the past year, I have exceeded the 80-h maximum weekly hour limit (*n* = 382)	
Frequently	11 (3)
Occasionally	31 (8)
Rarely	79 (21)
Never	261 (68)
During the past year, I failed to have 1 day per week free of duty (averaged over 4 wk) (*n* = 389)	
Frequently	14 (4)
Occasionally	16 (4)
Rarely	24 (6)
Never	335 (86)
Are there situations in which you believe it would be appropriate to violate duty hour rules? (*n* = 386)	
Yes	307 (80)
No	79 (20)
Have you ever been asked to violate duty hour rules? (*n* = 388)	
Yes	25 (6)
No	363 (94)
If so, by whom were you asked to violate duty hour rules? (*n* = 26)	
Peer	2 (8)
Senior resident	7 (27)
Attending physician	12 (46)
Other	5 (19)

*Abbreviation*: *ACGME* Accreditation Council for Graduate Medical Education

### Perceptions of fatigue, medical errors, and relation to duty hours

Most residents believed that fatigue (89%) and patient handovers (90%) contribute to adverse events (Table 4). A majority of residents believed DHRs reduced resident fatigue (80%) and reduced the incidence of residents performing clinical duties while fatigued (74%). Many residents believed that they (71%) and their colleagues (66%) were able to perceive when fatigue affected the care they provide. However, only 37% of residents believed that DHRs decreased medical errors, whereas 44% believed the standards had no effect on medical errors and 19% believed medical errors were increased by DHRs. More than half of respondents (62%) believed the standards decreased patient familiarity and continuity of care. Of respondents, 41% believed that patient handovers contributed more to adverse events than fatigue, compared with 27% who believed fatigue is the larger contributor.

**Table 4:** Resident Perceptions of Fatigue, Medical Errors, and Relation to Duty Hours

Survey Question	Respondents, No. (%)
I believe the current ACGME standards help reduce resident fatigue (*n* = 385)	
Strongly agree	61 (16)
Agree	248 (64)
Disagree	66 (17)
Strongly disagree	10 (3)
I believe that I can consistently tell when fatigue is adversely affecting my clinical performance (*n* = 386)	
Strongly agree	52 (13)
Agree	221 (57)
Disagree	102 (26)
Strongly disagree	11 (3)
I believe that my resident colleagues can consistently tell when fatigue is adversely affecting their clinical performance (*n* = 388)	
Strongly agree	22 (6)
Agree	234 (60)
Disagree	120 (31)
Strongly disagree	12 (3)
I believe the ACGME duty hour rules decrease the frequency of residents performing clinical duties while fatigued (*n* = 387)	
Strongly agree	38 (10)
Agree	250 (65)
Disagree	85 (22)
Strongly disagree	14 (4)
I believe the current ACGME standards ______ medical errors (*n* = 390)	
Markedly increase	15 (4)
Increase	61 (16)
Do not effect	171 (44)
Decrease	126 (32)
Markedly decrease	17 (4)
I believe the current ACGME standards __________ patient familiarity and continuity of care (*n* = 389)	
Markedly increase	7 (2)
Increase	26 (7)
Do not effect	116 (30)
Decrease	192 (49)
Markedly decrease	48 (12)
I believe that resident fatigue contributes to adverse events (*n* = 390)	
Strongly agree	112 (29)
Agree	235 (60)
Disagree	39 (10)
Strongly disagree	4 (1)
I believe that patient handovers contribute to adverse events (*n* = 390)	
Strongly agree	113 (29)
Agree	237 (61)
Disagree	38 (10)
Strongly disagree	2 (1)
Regarding the contribution of fatigue and handovers to adverse events, which of the following do you believe contribute more significantly to adverse events? (*n* = 389)	
Fatigue	105 (27)
Handovers	158 (41)
Both contribute equally	121 (31)
Neither contribute	5 (1)
During my training, I have made an error in patient care at the conclusion of an extended shift (>24 h) (*n* = 387)	
Yes	80 (21)
No	307 (79)
If so, did the error result in patient harm? (*n* = 100)	
Yes	10 (10)
No	90 (90)
During my training, I have been involved in a motor vehicle collision or potentially life-threatening event while leaving the hospital after an extended shift (>24 h) (*n* = 387)	
Yes	16 (4)
No	371 (96)

*Abbreviation*: *ACGME* Accreditation Council for Graduate Medical Education

### Perceptions of educational impact of duty hour requirements

Among residents, 26% indicated that DHRs enhanced their clinical educational experiences, whereas 33% believed the requirements had no effect and 41% felt the requirements diminished their clinical experiences (Table 5). When questioned about perceptions of duty hour effects on board examinations, academic productivity, and scheduled educational conferences, opinions were mixed. Opinions about the 16-h work limit for interns also varied; however, a majority of residents (73%) believed that their specialty should be subject to the same DHRs as other specialties.

**Table 5:** Resident Perceptions of Educational Impact of Duty Hour Requirements

Survey Question	Respondents, No. (%)
I believe the current ACGME standards ______ residents’ clinical educational experiences (*n* = 388)	
Markedly enhance	19 (5)
Enhance	81 (21)
Do not effect	127 (33)
Diminish	129 (33)
Markedly diminish	32 (8)
I believe the current ACGME standards __________ residents’ preparation for specialty board examinations (*n* = 386)	
Markedly enhance	12 (3)
Enhance	104 (27)
Do not effect	185 (48)
Diminish	67 (17)
Markedly diminish	18 (5)
I believe the current ACGME standards _________ residents’ academic productivity (publications, presentations, attendance and participation at national conferences) (*n* = 384)	
Markedly enhance	34 (9)
Enhance	157 (41)
Do not effect	152 (40)
Diminish	34 (9)
Markedly diminish	7 (2)
I believe the current ACGME standards _________ residents’ ability to attend daily or weekly educational conferences, such as didactics, morning report, Grand Rounds, and noon conferences (*n* = 387)	
Markedly enhance	28 (7)
Enhance	112 (29)
Do not effect	151 (39)
Diminish	84 (22)
Markedly diminish	12 (3)
I believe PGY-1 residents should work no more than 16 h of continuous duty (*n* = 386)	
Strongly agree	62 (16)
Agree	128 (33)
Disagree	133 (34)
Strongly disagree	63 (16)
I believe my specialty should be subject to the same duty hour regulations as residents in other specialties (*n* = 386)	
Strongly agree	78 (20)
Agree	205 (53)
Disagree	61 (16)
Strongly disagree	42 (11)

*Abbreviations*: *ACGME* Council for Graduate Medical Education, *PGY* postgraduate year

## Discussion

In this cross-sectional survey study of residents enrolled in all ACGME-accredited core residency programs at a large academic medical center, we found that a majority of residents were satisfied with the ACGME DHRs and perceived them as having an overall positive effect on their training.

Residents training in specialties categorized as *surgical* were significantly less likely to be satisfied with the DHRs or to report DHRs having a positive effect on their training than those categorized as *medical and pediatric* or *other*. Our results mirror an existing theme in the medical education literature, wherein concerns about the DHRs impact on surgical training in particular are highlighted [12, 15, 19–21]. Prior assessments of attitudes about DHRs by specialty have been reported, although among smaller samples and generally comparing 2 individual specialties. A multicenter survey study of 159 general surgery and internal medicine trainees from 3 centers demonstrated similar opinions about DHRs [22]. A more recent study of 202 general surgery and internal medicine residents and faculty compared a subset of 49 resident respondents and found that surgical residents favored DHRs significantly less than their internal medicine counterparts [17]. Our findings indicate that residents in specialties broadly categorized as *surgical* view the DHRs significantly less favorably than their colleagues in nonsurgical specialties. We chose not to report findings by individual specialty to ensure the confidentiality of respondents who are enrolled in programs with few trainees.

Residents in advanced stages of training, defined as PGY-3 and above, were also significantly less likely to be satisfied with DHRs or to report overall benefit to their training than those at a PGY-1 or 2 level. A national survey of family medicine residents similarly found that PGY-1 residents viewed DHRs more favorably than those in the PGY-2 year (25% vs 23%) [23]. That our finding is of greater magnitude may reflect the comparison of junior residents to those at considerably more advanced stages of training. The Flexibility in Duty Hour Requirements for Surgical Trainees (FIRST) trial, a prospective, randomized trial assessing impact of duty hour flexibility for general surgical trainees, also demonstrated increased concerns among senior residents, compared with junior residents, about negative effects of standard DHRs on patient safety and continuity of care [24].

Despite the differences we observed between the surgical versus nonsurgical disciplines and senior versus junior residents, it is worth stating that a majority of these subgroups did ultimately report overall satisfaction with the DHRs. Most respondents also indicated that the DHRs were respected and enforced by their institution. While most indicated that situations do exist where violation of DHRs is justified, few reported more than sporadic violations within the past year.

Although a majority of respondents believed the DHRs mitigate resident fatigue, only 37% believed that the requirements decreased medical errors. More than one-half of respondents believed that DHRs did not impact (44%) or increased (19%) medical errors. Our findings are congruent with a study using a grounded-theory analysis of resident comments, that found residents did not associate patient safety with DHR compliance [16, 25]. This may be in part due to a perception that patient handovers have increased and continuity of care has decreased. Our study does not directly address this question; however, we did find that a majority of residents believed that patient handovers contribute equally or more significantly to adverse patient events than fatigue. Arora and colleagues [10] found interns’ perceptions of the tradeoff between lack of care continuity and fatigue to depend largely on clinical context. Other studies have demonstrated that residents cite handovers as an important factor contributing to adverse events [15, 26], and existing empirical studies and national resident surveys have demonstrated a significant increase in the number of patient handovers following implementation of DHRs [13, 27].

Only 26% of residents perceived the DHRs to have a positive impact on their clinical educational experience. Prior studies have demonstrated findings of perceived reductions in bedside teaching, clinical educational experiences [22], and surgical opportunities [12, 20]. However, it is of importance to note that a large review of DHRs among surgical residents revealed no adverse effects on residents’ operative experience [28]. A subsequent systematic review associated the 16-h duty maximums with a nonsignificant trend toward worsened educational outcomes [21]. Similarly, the FIRST trial showed no significant difference between standard and flexible duty hour groups with regard to overall educational quality [24].

Strengths of our study are the large sample size and high resident response rate. In addition, we surveyed residents currently enrolled in all ACGME-accredited core residency programs at a large academic institution, and included residents at all levels of training within those programs. We also endeavored to conduct a thorough evaluation of the survey instrument by piloting our survey among experienced medical educators prior to its implementation.

Limitations of our study include a cross-sectional study design, single-institution sampling, and a subjective assessment by the study subject relating to Level 1 outcomes [29]. Because our survey was optional, it may also be limited by sampling bias. Despite the high response rate, it is possible that those who opted not to take the survey are dissimilar from those who chose to respond. Future work should focus on multi-institutional assessments that would allow for greater generalizability and an understanding of resident perceptions of DHRs across the specific individual specialties. Further, use of qualitative research methods, such as those used by Philibert [16], may prove useful in determining the underlying reasons for residents’ perceptions of DHRs [30].

## Conclusion

In this cross-sectional survey study of residents enrolled in all ACGME-accredited core residency programs at a large academic medical center, a majority of respondents were satisfied with the current ACGME DHRs and believed they affected their training in an overall positive manner. Residents in surgical specialties and those in advanced stages of training were significantly less likely to view DHRs favorably, although in these subgroups as well, a majority of the respondents did report overall satisfaction with the DHRs.

## Abbreviations

ACGME: Accreditation Council for Graduate Medical Education
DHR: Duty hour requirement
FIRST: Flexibility in Duty Hour Requirements for Surgical Trainees
med/peds: Medical and pediatric
PGY: Postgraduate year
REDCap: Research electronic data capture
